# MicroRNAs as Biomarkers for Acute Atrial Remodeling in Marathon Runners (The miRathon Study – A Sub-Study of the Munich Marathon Study)

**DOI:** 10.1371/journal.pone.0148599

**Published:** 2016-02-09

**Authors:** Sebastian Clauss, Reza Wakili, Bianca Hildebrand, Stefan Kääb, Eva Hoster, Ina Klier, Eimo Martens, Alan Hanley, Henner Hanssen, Martin Halle, Thomas Nickel

**Affiliations:** 1 Medizinische Klinik und Poliklinik 1, Campus Grosshadern, Ludwig-Maximilians-Universität München (LMU), Munich, Germany; 2 DZHK (German Centre for Cardiovascular Research), Partner site Munich, Munich Heart Alliance, Munich, Germany; 3 Cardiovascular Research Center, Massachusetts General Hospital, Charlestown, MA, United States of America; 4 Institute for Medical Informatics Biometry and Epidemiology, Ludwig-Maximilians-UniversitätMünchen, Munich, Germany; 5 Department of Prevention and Sports Medicine, TechnischeUniversitätMünchen, Klinikumrechts der Isar (MRI), Munich, Germany; 6 Sports Medicine, Institute of Exercise and Health Sciences, University Basel, Basel, Switzerland; IPMC, CNRS UMR 7275 UNS, FRANCE

## Abstract

**Introduction:**

Physical activity is beneficial for individual health, but endurance sport is associated with the development of arrhythmias like atrial fibrillation. The underlying mechanisms leading to this increased risk are still not fully understood. MicroRNAs are important mediators of proarrhythmogenic remodeling and have potential value as biomarkers in cardiovascular diseases. Therefore, the objective of our study was to determine the value of circulating microRNAs as potential biomarkers for atrial remodeling in marathon runners (miRathon study).

**Methods:**

30 marathon runners were recruited into our study and were divided into two age-matched groups depending on the training status: elite (ER, ≥55 km/week, n = 15) and non-elite runners (NER, ≤40 km/week, n = 15). All runners participated in a 10 week training program before the marathon. MiRNA plasma levels were measured at 4 time points: at baseline (V1), after a 10 week training period (V2), immediately after the marathon (V3) and 24h later (V4). Additionally, we obtained clinical data including serum chemistry and echocardiography at each time point.

**Results:**

MiRNA plasma levels were similar in both groups over time with more pronounced changes in ER. After the marathon miR-30a plasma levels increased significantly in both groups. MiR-1 and miR-133a plasma levels also increased but showed significant changes in ER only. 24h after the marathon plasma levels returned to baseline. MiR-26a decreased significantly after the marathon in elite runners only and miR-29b showed a non-significant decrease over time in both groups. In ER miRNA plasma levels showed a significant correlation with LA diameter, in NER miRNA plasma levels did not correlate with echocardiographic parameters.

**Conclusion:**

MiRNAs were differentially expressed in the plasma of marathon runners with more pronounced changes in ER. Plasma levels in ER correlate with left atrial diameter suggesting that circulating miRNAs could potentially serve as biomarkers of atrial remodeling in athletes.

## Introduction

There is compelling evidence that physical activity has beneficial effects, especially regarding cardiovascular health[1]. It has been shown that exercise can reduce the risk of stroke[2], coronary heart disease[3], atherosclerosis[4], or heart failure[5]. Continuous exercise induces physiological adaptation including cardiac enlargement and mild left ventricular hypertrophy, classically characterized as “athlete’s heart”[6, 7]. In contrast, it has also been demonstrated that participation in endurance sports is associated with increased risk of disease[8], in particular arrhythmias like sinus node dysfunction[9], heart block[10], or atrial fibrillation[11].One explanation for these are underlying cardiac pathologies such as cardiomyopathy. However an increasing body of evidence suggests that exercise-induced remodeling processes are not purely benign but can also create an arrhythmogenic substrate.

Benito et al. demonstrated that daily treadmill exercise in rats for 16 weeks resulted in eccentric ventricular hypertrophy, diastolic dysfunction, atrial enlargement and increased myocardial fibrosis [12]. They could also induce ventricular tachyarrhythmias in 42% of these rats (vs. 6% in control rats). Guasch et al. observed a significantly increased inducibility of AF in the same rat model (64% vs. 15% after 16 weeks of 1h/d treadmill exercise)[13]. They demonstrated autonomic dysregulation as well as significant left atrial dilatation and atrial fibrosis, neither of which recovered fully with exercise cessation. Irreversible acute atrial remodeling caused by endurance exercise might therefore be a potential explanation for long-term morbidity in athletes.

In experimental animal models it is easy to measure molecular, genomic, or cellular changes. However, in humans we have to use surrogate parameters to estimate atrial remodeling[14]. One of the hallmarks of atrial remodeling that can be measured by echocardiography is left atrial dilatation[15]. LA dilatation is a suitable surrogate parameter for atrial remodeling as it is associated with atrial fibrosis, reduced atrial function, and increased risk for AF development[16–18]. Another surrogate parameter is the peak A wave velocity measured by pulsed wave Doppler echocardiography that has been widely used to assess atrial function[19–22] and has been shown to be associated with AF risk in the Framingham Heart Study and the Cardiovascular Health Study[17, 23]. Furthermore, mitral annular E/E’ has been reported as an appropriate parameter to estimate the degree of atrial remodeling[24] since diastolic dysfunction is an independent risk factor for AF[25, 26]. Additionally, it has been shown that recurrence of AF after electrical cardioversion can be predicted by the degree of diastolic dysfunction[27] or LA dilatation[28].

In recent years, microRNAs (miRNAs) have been shown to play an important role in AF pathophysiology by regulating remodeling processes[29–34]. MiRNAs are short, single stranded, and non-coding RNA fragments that bind to the 3’ UTR of their target genes leading to inhibition of mRNA translation. Therefore, miRNAs are post-transcriptional regulators of gene expression either by direct inhibition (binding to the 3’UTR of the target gene) or indirect activation (binding to the 3’UTR of an endogenous inhibitor). MiRNAs have been shown to play an important role in atrial remodeling. MiR-1 and miR-26a are implicated in electrical remodeling by regulating ion channels[35–37] or calcium homeostasis[38]. MiR-29b, miR-30a and miR-133a are predominantly involved in structural remodeling causing enhanced atrial fibrosis [31, 39].

Recently, several studies have shown that endurance sport and aerobic exercise impact on the level of circulating miRNAs[40, 41]. Mooren and colleagues evaluated miRNA plasma levels in marathon runners and demonstrated that miR-1, miR-206 and miR-133a plasma levels are increased after a marathon and are associated with aerobic performance parameters[41]. Baggish et al. performed a study in marathon runners and found that miR-1, miR-133a, miR-126, miR-134, miR-146a, miR-208a, and miR-499 were differentially regulated[40]. In their study the plasma profile of miRNAs and conventional cardiac injury markers like troponin differed suggesting a potential role for miRNAs as biomarkers for exercise-induced cardiac adaptation.

In order to determine the potential value of miRNAs as biomarkers for acute atrial remodeling in athletes we performed the miRathon study, analyzing the plasma profile of 5 miRNAs associated with atrial remodeling in marathon runners over time.

## Materials and Methods

### Study design

Our study was designed as a sub-study of the previously published Munich Marathon study[42, 43]. In brief, 30 marathon runners intending to participate in the Munich Marathon were recruited via a local newspaper and by written invitations sent to local running clubs. Recruitment was limited to healthy male marathon runners aged 30–60 years who had run at least a half-marathon in the previous 3 years and who had no cardiovascular risk factors. The candidates volunteered for an individually tailored, supervised training program. The group was divided into two age-matched groups depending on the training status: elite runners (ER) and non-elite runners (NER). ER performed regular intensive exercise throughout the year and were scheduled for ≥55 km/week during the 10 week training program. The NER group was scheduled for ≤40 km/week with only seasonal pre-marathon exercise training. The 10 week endurance exercise program was according to current guidelines[44]. Before and after the training program each runner performed a symptom-limited treadmill ergometry to determine the individual anaerobic threshold (IAT) and to quantify the individual fitness improvement. Blood was collected at baseline (V1), after a 10 week training period (V2), immediately after the marathon (V3), and 24 hours later (V4).

### Blood collection

Fasting blood samples were taken 2–5 days before the marathon and immediately after the marathon. Runners did not exercise during the two days prior to baseline blood sampling. Blood samples were collected via direct venous puncture into 9 ml EDTA containing tubes (SarstedtMonovette). All blood was processed for isolation of plasma within 4 hours of collection. Blood was processed by spinning at 4000 rpm for 20 minutes at room temperature. Plasma was carefully transferred to a fresh RNAse/DNAse free tube and stored at -80°C.

### RNA isolation

RNA isolation and miRNA plasma level measurement was performed as previously described[31]. In brief, plasma was thawed on ice and 400 μL EDTA-plasma was mixed with 4000 μLTRIzol (Invitrogen), incubated for 5 minutes at room temperature and subsequently mixed with 800 μL chloroform. The organic and aqueous phases were separated by centrifugation. The aqueous phase containing the RNA was carefully removed and RNA was precipitated by addition of 100% ethanol. The mixture was applied to an RNeasy Mini spin column (Qiagen), washed several times and RNA was eluted by addition of 35 μL RNase-free water (95°C).

As no plasma housekeeping miRNA in the context of exercise has been established and validated to normalize for the miRNA content to date, we chose to use a fixed volume of plasma per sample and a synthetic Caenorhabditis elegans miR-39 (cel-miR-39, 20 fmol/sample, synthesized by Qiagen) as a spiked-in control to normalize for individual RNA-isolation-related variations. Twenty fmol cel-miR-39 were introduced to each plasma sample after addition of denaturatingQiazol solution. For each RNA sample, the C. elegans spiked-in miRNAs were measured using TaqManqRT-PCR assays (Applied Biosystems).

### Hemolysis assessment

To assess the degree of hemolysis we measured absorbance at 414 nm using a NanoDrop1000 as described elsewhere[45]. Values above 0.2 were indicative of hemolysis.

### Measurement of miRNA Levels in Plasma with TaqMan qPCR Assays

A fixed volume of diluted RNA (5 μL) was subjected to reverse transcription using the TaqMan microRNA Reverse Transcription kit (Applied Biosystems) according to the manufacturer’s protocol. Subsequently, 1.33 μL of the product was used to detect miRNA-expression by quantitative PCR using miRNA-specific stem-loop primers (Applied Biosystems) for the corresponding microRNA. Quantitative PCR reactions were performed on a Bio-Rad iQ5 system using the following program: 10 minutes pre-incubation at 95°C, 45cycles of 15 seconds denaturation at 95°C and 60 seconds of elongation at 60°C. Values are normalized to cel-miR-39 and expressed as 2^-[(CT microRNA)-(CT cel-miR-39)]^.

### Echocardiography

All studies were performed using a commercially available echocardiography device equipped with a 2.5-MHz probe and digital storage capacity (Philips iE32 System; PhilipsHealthcare). Inter-observer variability was eliminated by having all studies performed and analysed by a single experienced investigator. To facilitate imaging of all participants immediately postrace, the echocardiographyexam was limited to measurements of LV systolic and diastolic function. The study was performed in two-dimensional (2D) and colour tissue Doppler (TD) imaging modes. 2D measurements included LV end-diastolic and end-systolic volumes. Systolic ejection fraction was calculated using Simpson’s rule (biplane). Cardiac size was determined by assessing total LV end-diastolic volume per kg of body weight (TEDD3/kg), as previously described in athletes. Pulsed mitral annular Doppler and colour TD were used to determine regional and global diastolic function. Mitral inflow velocities E and A and colour TD measurements of septal mitral annulus velocities E’ and A´ were performed in the apical four-chamber view[46].

### Measurement of Troponin T, CK, and CK-MB

Troponin T was quantified using electrochemiluminescence sandwich immunoassay (Roche, Switzerland) according to the manufacturer’s recommendations. Serum CK activity was quantified using a Beckman Coulter analyser system AU5800 (Beckman Coulter Inc., Brea CA, USA) based on a photometric test according to the International Federation of Clinical Chemistry (IFCC method). For the quantification of CK-MB an immuno-inhibition method on the same analytical routine platform was used.

### Statistical analysis

Data are presented as MEAN±SEM. GraphPad Prism 5.01 was used for statistical analysis. Friedman-Test was used for multi-group comparisons. Spearman’s correlation analysis was used to identify any significant relationships. A p<0.05 was considered statistically significant.

### Ethics statement

The study was approved by the hospital’s ethics committee of the Technical University of Munich. Investigations were performed according to the 1975 Declaration of Helsinki. Written informed consent was obtained prior to study enrolment by all participants.

## Results

### Clinical characteristics

In our study we evaluated 2 age-matched groups of athletes participating in the Munich marathon: elite runners (n = 15) who performed regular endurance exercise throughout the year and non-elite runners (n = 15) without regular endurance exercise.

Since our study was designed as a sub-study of the Munich Marathon Study, marathon performance data has already been published elsewhere[42, 43]. In brief, during the training period elite runners ran 73.9±3.9 km per week whereas non elite runners ran 33.9±2.7 km per week (*** p<0.001, Table 1). Before the 10 week training program baseline heart rate was significantly lower in elite runners (50.5±2.4 vs. 61.5±2.9, ** p = 0.006) and IAT was significantly higher in elite runners (13.4±0.4 vs. 11.9±0.3, ** p = 0.001). 10 weeks of training resulted in a marked improvement of both training parameters. IAT was significantly increased and resting heart rate was decreased in both groups with more pronounced changes in non-elite runners (Table 1). None of the performance parameters correlated with miRNA plasma levels.

**Table 1:** Clinical characteristics.

		pre training			post training	
	Elite runners	Non-elite runners	p	Elite runners	Non-elite runners	p
**demographic data**						
*age (years)*	40.0±1.7	40.1±1.4	0.953			
**training parameters**						
*BMI*	23.3±0.55	24.1±0.42	0.222	22.8±0.55	23.7±0.45	0.213
*resting heart rate (/min*.*)*	50.5±2.39	61.5±2.85	**0.006** **	50.1±2.09	58.1±2.36	**0.018** *
*km per week*	73.9±3.86	33.9±2.72	**0.000007** ***			
*IAT (km/h)*	13.4±0.36	11.9±0.25	**0.001** **	13.6±0.363	12.4±0.25	**0.011** *
*heart rate at IAT (/min*.*)*	168.7±2.97	167.1±2.54	0.675	167.4±2.91	164.9±2.05	0.494
*lactat (mmol/l)*	2.8±0.156	3,09±0.199	0.187	2.6±0.12	2.8±0.19	0.520
*systolic blood pressure (mmHg)*	127.3±2.48	127.7±3.93	0.943	128.0±2.79	132.0±3.44	0.375
*diastolic blood pressure (mmHg)*	79.0±1.11	79.0±2.14	1.000	83.0±1.88	85.7±2.17	0.361
**Echocardiography**						
*Ejection fraction (%)*	65.9±1.04	59.6±4.58	0.252	65.8±0.94	60.0±4.55	0.513
*LA diameter (mm)*	38.9±1.26	35.5±2.69	1.000	38.1±1.28	35.1±2.72	0.871
*LVEDD (mm)*	50.2±1.05	46.9±3.50	0.796	49.2±1.18	46.5±3.60	0.568
*posterior wall thickness (mm)*	11.5±0.34	9.5±0.88	0.181	12.5±0.46	10.7±0.91	0.905
*E wave velocity (cm/s)*	83.7±3.26	73.0±6.57	0.189	82.1±4.06	69.8±6.55	0.130
*A wave velocity (cm/s)*	46.3±2.59	44.2±4.26	0.588	54.7±3.13	47.5±4.41	0.385
*E/E‘ ratio*	7.8±0.26	7.0±0.67	0.838	8.7±0.30	7.5±0.67	0.285

BMI: body mass index.

km: kilometer.

IAT: individual aerobic threshold.

LA: left atrium.

LVEDD: left ventricular end-diastolic diameter.

*p<0.05

**p<0.01

***p<0.001 elite vs. non-elite runners.

### MicroRNA plasma level

**Fig 1:**
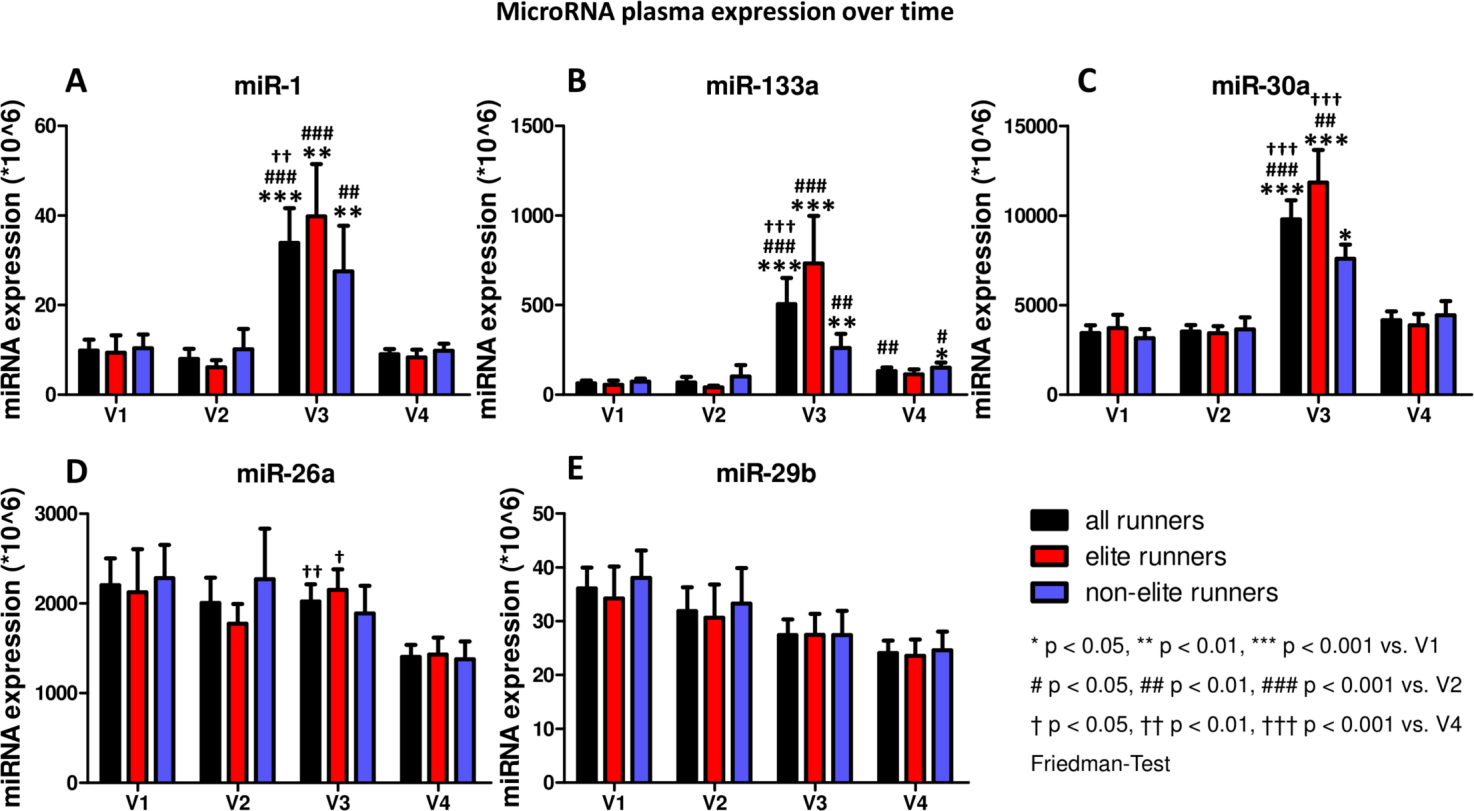
MicroRNA plasma expression over time. A miR-1, B miR-133a, C miR-30a, D miR-26a, E miR-29b. V1 baseline, V2 after a 10 week training period, V3 immediately after the marathon, V4 24 hours after the marathon. Data shown as MEAN±SEM, * p < 0.05, ** p<0.01, *** p<0.001 vs. timepoint V1, # p < 0.05, ## p<0.01, ### p<0.001 vs. timepoint V2, † p < 0.05, †† p<0.01, ††† p<0.001 vs. timepoint V4, Friedman-Test.

MicroRNA plasma levels at baseline (V1) were similar in both groups (Table 2, Fig 1). The 10 week training program had no significant impact on the miRNA plasma level (V2). At the end of the marathon (V3) miR-1, miR-133a and miR-30a showed a significant increase (*** p<0.001 vs. before marathon (V2)). However, this increase was mainly driven by the changes in elite runners. 24 hours after the marathon (V4) plasma levels of miR-1, miR-133a and miR-30a decreased significantly.

**Table 2:** MiRNA raw CT values. Data shown as raw cycle numbers (Min-Max).

miRNA	Assay-ID	Baseline (V1)	After 10 weeks training (V2)	directly after the marathon (V3)	24h post marathon (V4)
miR-1	000385	34,94–40,64	34,96–42,37	32,89–43,17	35,50–39,74
miR-26a	000405	34,96–39,65	34,36–40,39	33,70–41,33	35,08–39,39
miR-29b	000413	33,76–36,59	33,13–38,42	34,43–39,48	34,57–36,85
miR-30a	000416	34,95–39,65	34,76–41,05	33,43–41,94	35,22–39,39
miR-133a	002246	34,96–39,65	34,36–40,39	33,70–41,33	35,08–39,39

MiR-26a and miR-29b showed a different expression pattern in plasma over time. After the marathon (V3) both miRNAs were downregulated and showed a further decrease 24 hours later (V4). However, only miR-26a was significantly downregulated in elite runners only (V4 compared to V3) whereas non-elite runners and miR-29b levels in both group showed a non-significant trend towards downregulation (Fig 1).

### Assessment of hemolysis

**Fig 2:**
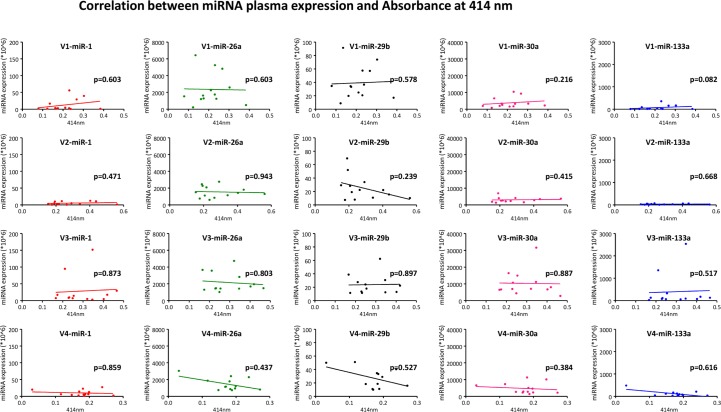
Correlation between miRNA plasma expression and Absorbance at 414 nm. Different time points (V1-V4) shown from top to bottom, different miRNAs (miR-1, -26a, -29b, -30a, -133a) are shown from left to right. Spearman correlation coefficient.

Since hemolysis can affect the level of miRNAs in plasma samples we performed a quality control by measuring absorbance at 414 nm on 13 samples at each time point. Although measurements indicated some degree of hemolysis (Table 3) in some samples we could not detect any correlation between the degree of hemolysis (as indicated by the absorbance) and the miRNA expression level (Fig 2).

**Table 3:** Hemolysis Assessment.

	Absorbance at 414 nm			
participant	Baseline (V1)	After 10 weeks training (V2)	directly after the marathon (V3)	24h post marathon (V4)
03	0,211	0,342	0,232	0,188
05	0,303	0,403	0,419	0,238
06	0,231	0,235	0,174	0,17
08	0,267	0,437	0,347	0,181
09	0,135	0,154	0,211	0,031
17	0,175	0,207	0,229	0,197
25	0,389	0,575	0,466	0,285
39	0,172	0,177	0,254	0,148
47	0,238	0,264	0,346	0,185
50	0,079	0,19	0,166	0,116
57	0,23	0,217	0,402	0,168
60	0,123	0,194	0,249	0,199
61	0,162	0,292	0,321	0,183

### Skeletal muscle and cardiac serum markers

Serum levels of troponin T (published before[43]), CK and CK-MB were used in our study for calculation of potential correlations with miRNA plasma levels (Table 4). Only 2 measurements of CK-MB at visit 3 (both elite runners) did not pass our internal quality control and were therefore not used for further calculation.

**Table 4:** Skeletal muscle and cardiac serum markers.

		V1			V2			V3			V4	
	Elite runners	Non elite runners	p	Elite runners	Non elite runners	p	Elite runners	Non elite runners	p	Elite runners	Non elite runners	p
**CK (U/l)**	220.0±24.5	277.1±59.2	0.381	178.9±21.4	174.5±26.6	0.901	524.9±64.4	531.3±103.3	0.959	1986.7±326.5	1928.4±423.3	0.914
**CK-MB (U/l)**	23.2±4.8	23.0±5.4	0.983	12.5±10.2	20.0±3.5	0.522	30.1±3.9	25.5±2.3	0.343	62.6±16.5	47.3±6.2	0.421

In brief, serum levels of troponin T, CK and CK-MB were similar before (V1) and after the 10 week training program (V2) without any significant differences between groups. Serum levels of creatine kinase (CK) showed a non-significant trend towards higher levels after the marathon (V3) but increased significantly 24 hours later (V4). The serum levels of the MB isoform of creatine kinase (CK-MB) showed a similar pattern as CK but significant changes occurred only in non-elite runners 24 hours after the marathon (V4).

Troponin levels were below detection limit before the marathon (V1, V2), increased after the marathon (V3) significantly, and returned to non-measurable levels 24 hours later (V4). None of these markers differed significantly between elite and non-elite runners.

### Echocardiography

We obtained echocardiographic measurements from all 30 participants at all four time points. However, at visit 3 adequate measurement of LA diameter in 2 participants (both elite runners) was not possible.

**Fig 3:**
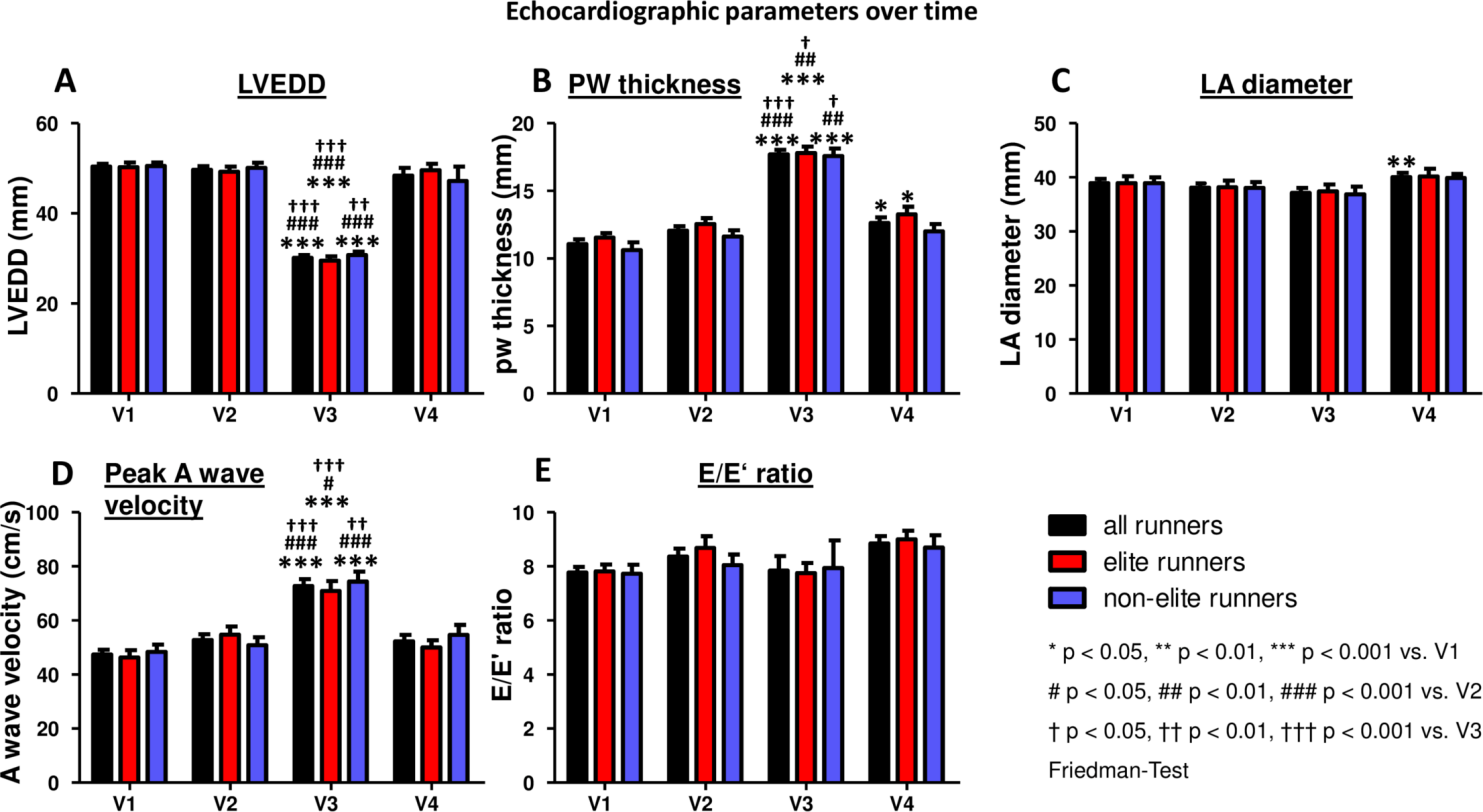
Echocardiographic parameters over time. A Left ventricular end-diastolic diameter (LVEDD), B Posterior Wall (PW) thickness, C Left atrial (LA) diameter, D Peak A Wave velocity, E E/E’ ratio. V1 baseline, V2 after a 10 week training period, V3 immediately after the marathon, V4 24 hours after the marathon. Data shown as MEAN±SEM, * p < 0.05, ** p<0.01, *** p<0.001 vs. timepoint V1, # p < 0.05, ## p<0.01, ### p<0.001 vs. timepoint V2, † p < 0.05, †† p<0.01, ††† p<0.001 vs. timepoint V3, Friedman-Test.

At baseline (V1)there was no significant difference between groupswith regard to left ventricular end-diastolic diameter (LVEDD), posterior wall (PW) thickness, left atrial (LA) diameter, peak A wave velocity, or E/E’ ratio (Fig 3). After the 10 week training program (V2) no significant changes were observed. After the marathon (V3) LVEDD was significantly reduced (*** p<0.001 vs. V2; Fig 3A), PW thickness and peak A wave velocity were significantly increased (*** p<0.001 vs. V2; Fig 3B and 3D), LA diameter showed a non-significant trend towards lower dimensions (Fig 3C) and E/E’ ratio showed no significant changes compared to baseline (Fig 3E). 24 hours after the marathon all parameters returned to a level similar to baseline. However, PW thickness, LA diameter, peak A wave velocity, and E/E’ ratio were still slightly increased (not significantly), whereas LVEDD was still slightly decreased (not significantly).

### Correlation between miRNA plasma levels and LA diameter

**Fig 4:**
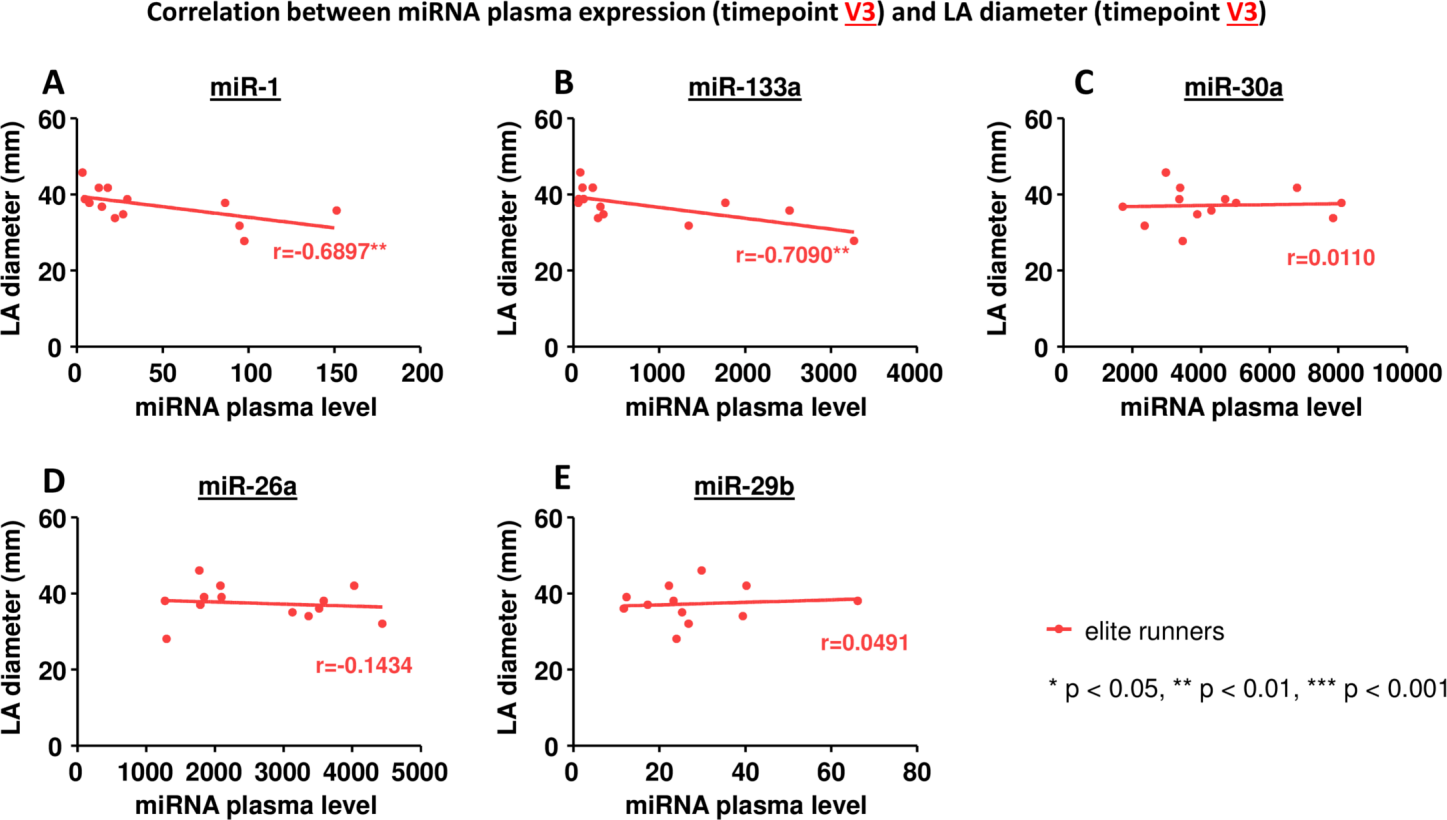
Correlation between miRNA plasma expression at timepoint V3 and LA diameter at timepoint V3. A miR-1, B miR-133a, C miR-30a, D miR-26a, E miR-29b. * p< 0.05, ** p<0.01, *** p<0.001 Spearman correlation coefficient.

**Fig 5:**
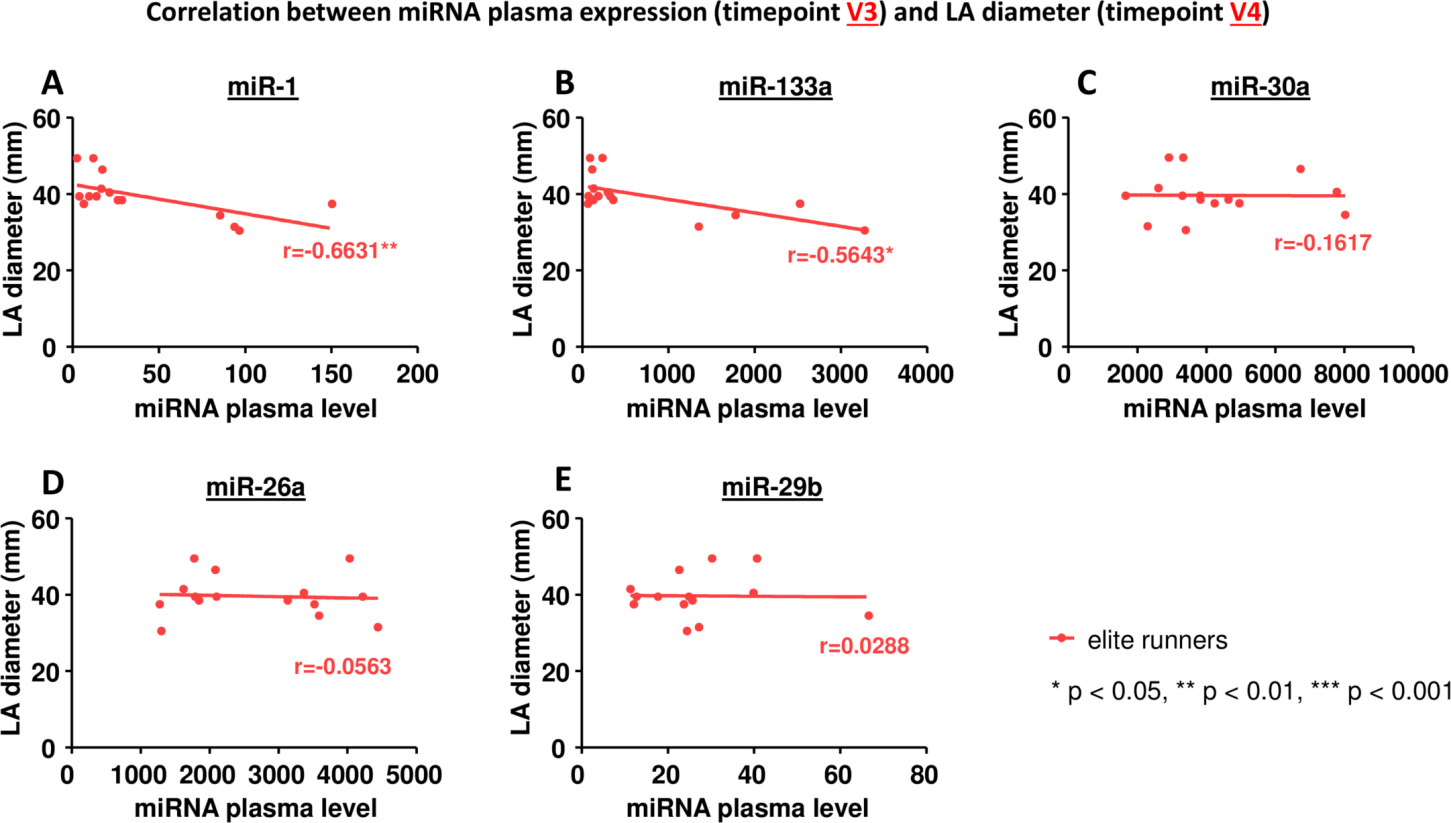
Correlation between miRNA plasma expression at timepoint V3 and LA diameter at timepoint V4. A miR-1, B miR-133a, C miR-30a, D miR-26a, E miR-29b. * p< 0.05, ** p<0.01, *** p<0.001 Spearman correlation coefficient.

We could not find any correlation between miRNA plasma levels before the marathon (V2) and LA diameter before (V2) or after the marathon (V3). However, we observed a significant correlation between the peak plasma levels of miR-1 and miR-133a and LA diameter after the marathon (V3) in elite runners (Fig 4, Table 5). Furthermore, peak plasma levels of miR-1 and miR-133a also correlated with LA diameter 24 hours after the marathon (V4; Fig 5, Table 5). In non-elite runners no correlation between miRNA plasma levels and LA diameter could be found (Table 6).

**Table 5:** Correlation analyses between miRNAs and clinical parameters in elite runners (ER).

		miR-1		miR-133a		miR-30a		miR-26a		miR-29b	
		r	p-value	r	p-value	r	p-value	r	p-value	r	p-value
miRNA plasma levels at V3	Peak A wave velocity at V3	0.1518	0.6044	0.0088	0.9762	0.1364	0.6419	-0.0792	0.7878	-0.3961	0.1802
	E/E’ at V3	-0.1610	0.5665	-0.0411	0.8842	-0.3417	0.2126	-0.2200	0.4307	-0.0308	0.9167
	LA diameter at V3	-0.6897	**0.0091**	-0.7090	**0.0067**	0.0110	0.9715	-0.1434	0.6401	0.0491	0.8861
	LA diameter at V4	-0.6631	**0.0070**	-0.5643	**0.0284**	-0.1617	0.5647	-0.0563	0.8587	0.0288	0.9222
	CK plasma levels at V3	0.7143	**0.0028**	0.7536	**0.0012**	0.5286	**0.0428**	0.1786	0.5243	0.3802	0.1799
	Troponin levels at V3	0.0841	0.7657	0.3234	0.2397	0.1701	0.5444	-0.1739	0.5355	0.3747	0.1868
	CK-MB levels at V3	0.7455	**0.0034**	0.8363	**0.0004**	0.6080	**0.0275**	0.2311	0.4475	0.0736	0.8171

**Table 6:** Correlation analyses between miRNAs and clinical parameters in non-elite runners (NER).

			miR-1		miR-133a		miR-30a		miR-26a		miR-29b	
			r	p-value	r	p-value	r	p-value	r	p-value	r	p-value
miRNA plasma levels at V3	Peak A wave velocity at V3		-0.1341	0.6477	-0.2352	0.4183	0.3022	0.3156	0.0550	0.8520	0.0945	0.7479
	E/E’ at V3		0.2398	0.4089	0.3124	0.2768	-0.2283	0.4531	-0.2266	0.4359	-0.0595	0.8400
	LA diameter at V3		0.2151	0.4602	0.0599	0.8389	0.0498	0.8717	0.1308	0.6557	0.0732	0.8037
	LA diameter at V4		0.5011	0.0679	0.2129	0.4650	0.1221	0.6912	-0.09535	0.7458	0.1020	0.7286
	CK plasma levels at V3		0.5473	**0.0428**	0.7231	**0.0035**	-0.2527	0.3833	-0.4330	0.1220	-0.3363	0.2398
	Troponin levels at V3		0.6316	**0.0154**	0.6603	**0.0102**	0.2010	0.4909	-0.2488	0.3910	-0.2010	0.4909
	CK-MB levels at V3		0.2564	0.4697	0.5065	0.1334	-0.1563	0.6567	0.0000	1.000	-0.2064	0.5603

We also evaluated potential correlations between miRNA plasma expression and other echocardiographic parameters but we could not observe any correlation between miRNA plasma levels and peak A wave velocity or E/E’ in either elite or non-elite athletes (Tables 5 and 6).

### Correlation between miRNA plasma levels and markers of muscle injury

**Fig 6:**
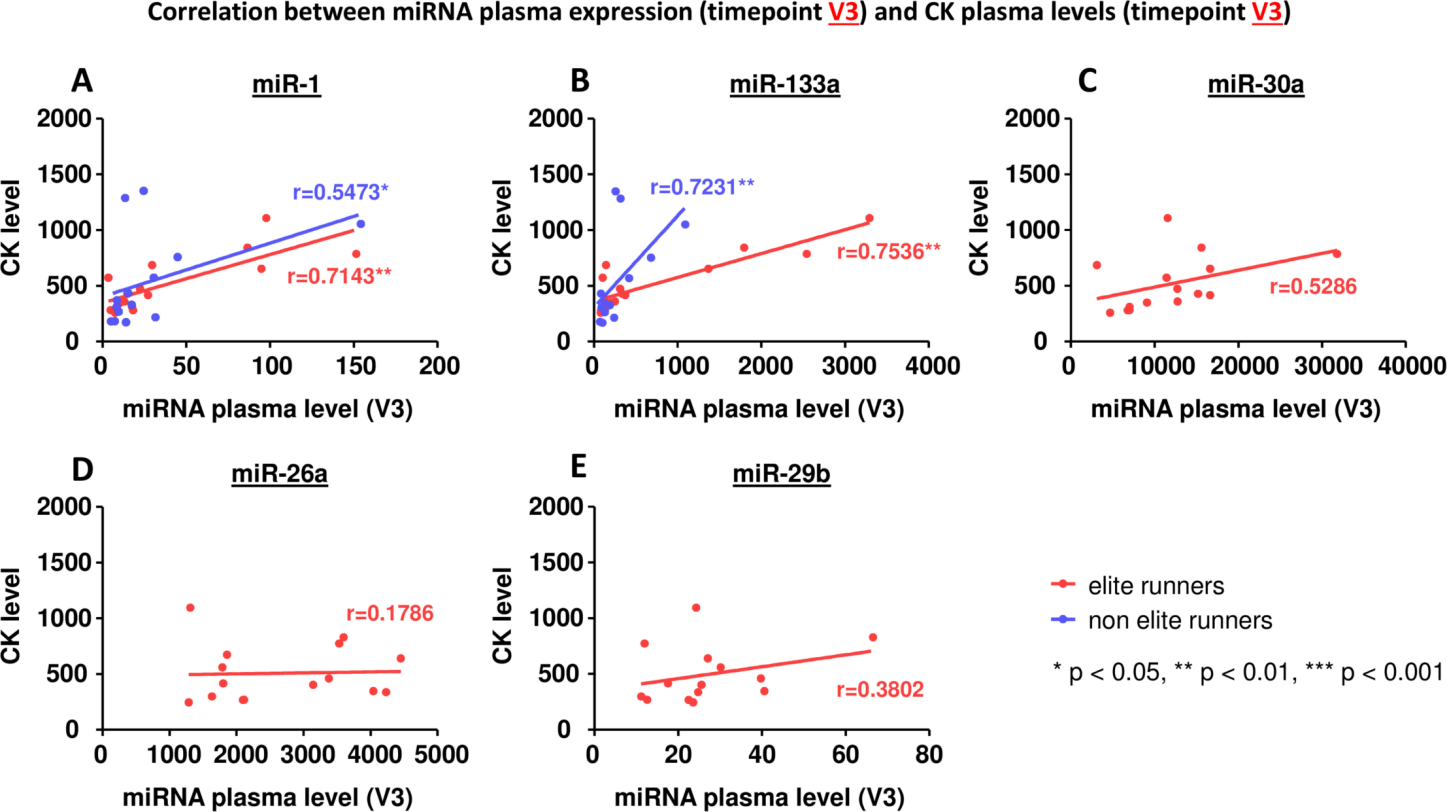
Correlation between miRNA plasma expression at timepoint V3 and plasma levels of creatine kinase at timepoint V3. A miR-1, B miR-133a, C miR-30a, D miR-26a, E miR-29b. * p< 0.05, ** p<0.01, *** p<0.001 Spearman correlation coefficient.

**Fig 7:**
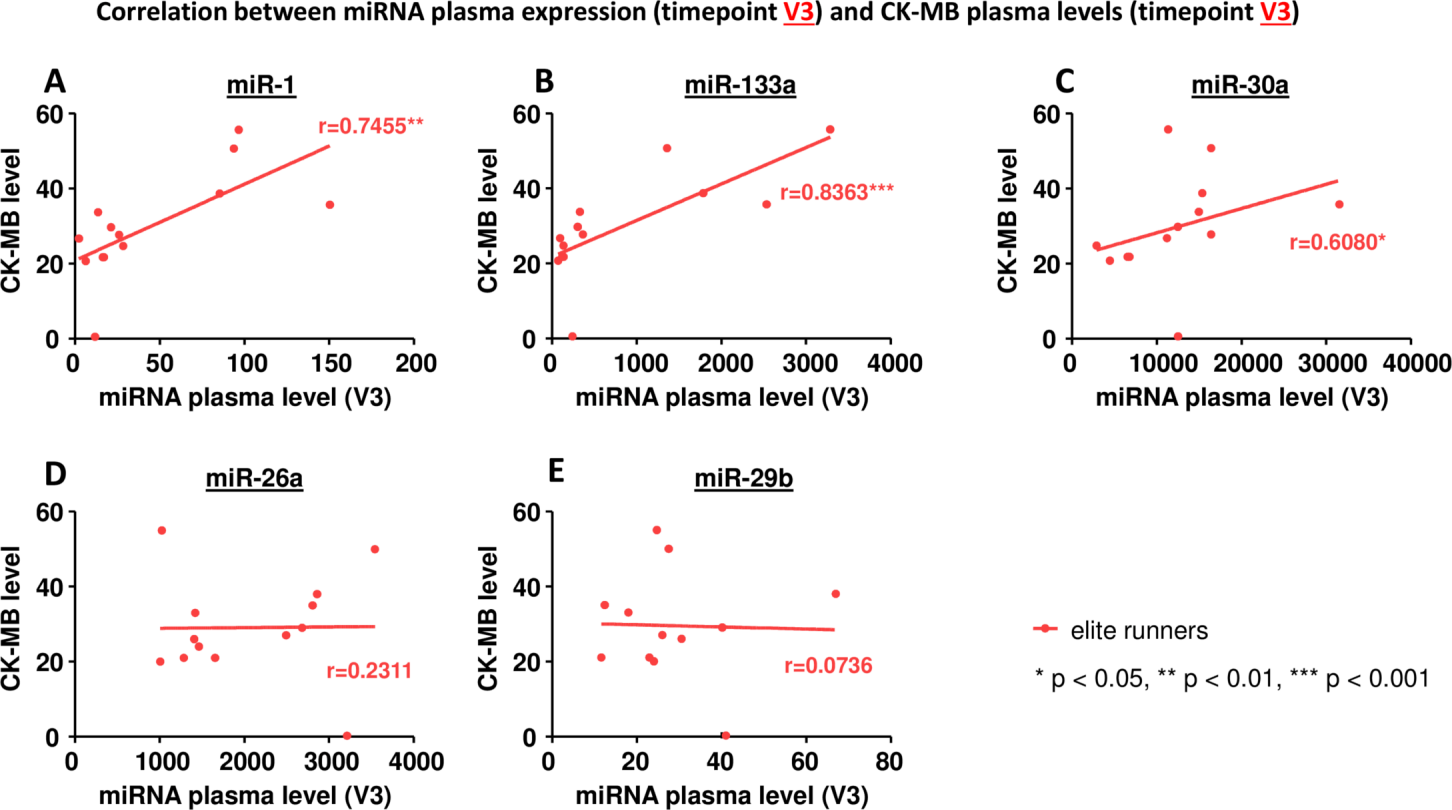
Correlation between miRNA plasma expression at timepoint V3 and plasma levels of creatine kinase, isoform MB at timepoint V3. A miR-1, B miR-133a, C miR-30a, D miR-26a, E miR-29b. * p< 0.05, ** p<0.01, *** p<0.001 Spearman correlation coefficient.

**Fig 8:**
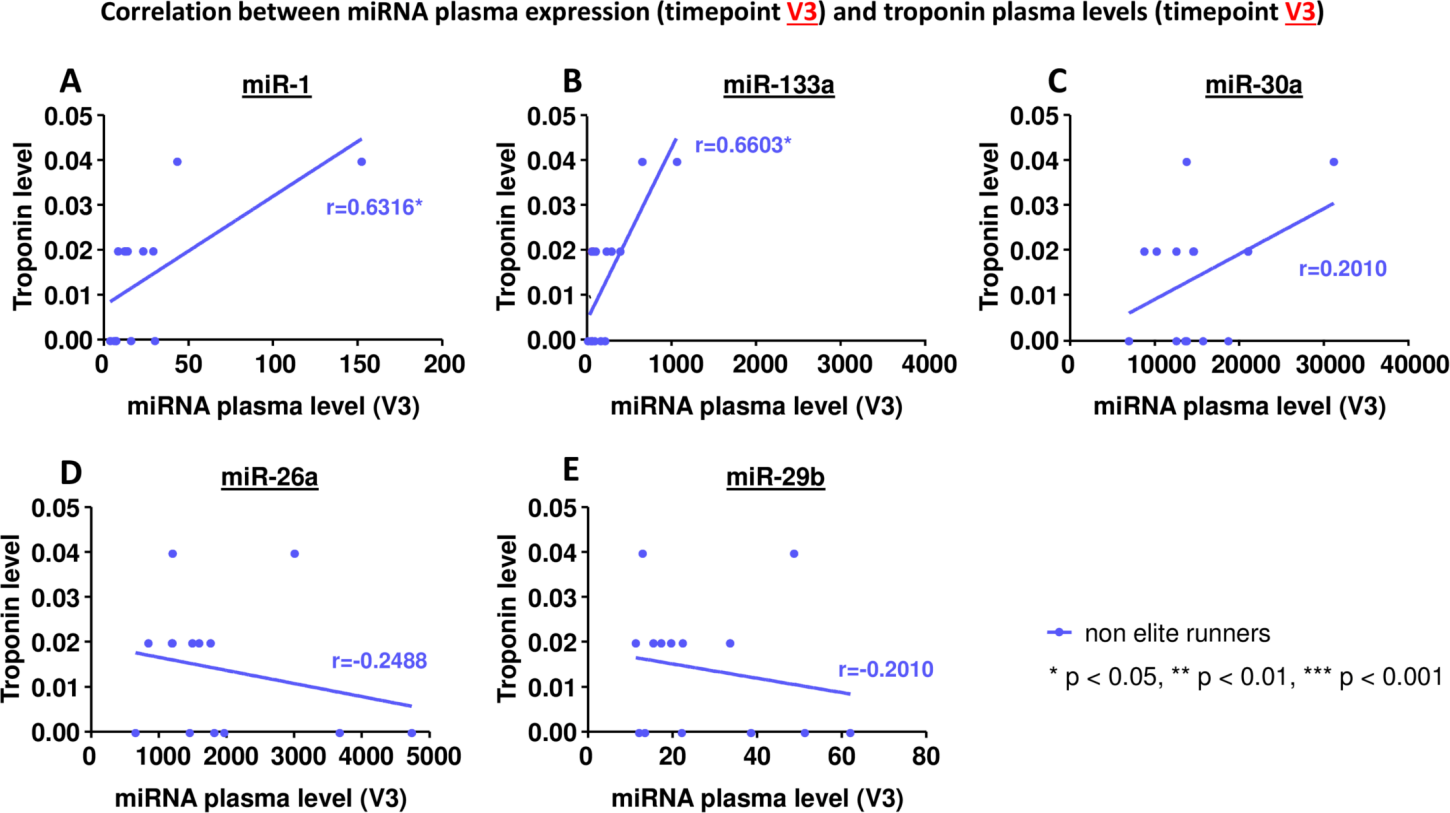
Correlation between miRNA plasma expression at timepoint V3 and plasma levels of troponin T at timepoint V3. A miR-1, B miR-133a, C miR-30a, D miR-26a, E miR-29b. * p< 0.05, ** p<0.01, *** p<0.001 Spearman correlation coefficient.

An increase in circulating miRNAs could potentially be caused by injured cells releasing their intracellular miRNAs into the blood. Therefore we evaluated if the miRNA plasma levels correlated with markers of cellular injury (Figs 6–8).

MiR-30a, miR-26a and miR-29b did not correlate with levels of creatine kinase, the MB isoform of creatine kinase, or troponin T (Figs 6–8, C-E). MiR-1 and miR-133a, however, showed a significant correlation: in elite runners these miRNAs correlate with creatine kinase and the MB isoform of it (Figs 6A and 6B, 7A and 7B). In non-elite runners miR-1 and miR-133a correlate with troponin T levels (Fig 8A and 8B).

## Discussion

In our miRathon study we measured circulating miRNAs in plasma and evaluated their potential value as biomarkers for atrial remodeling in marathon runners. We demonstrated a significant increase of miR-1, miR-30a and miR-133a immediately after the marathon with return to baseline 24 hours later. Furthermore, miR-26a and miR-29b showed a trend towards progressively reduced expression over time. In elite runners plasma miRNA levels after the marathon correlated with LA diameter, a parameter of structural remodeling.

### miRNAs as potential biomarkers

MiRNA biology has been a very active area of research recently, and several studies have been published evaluating circulating miRNAs as potential biomarkers of heart disease including acute infarction, coronary artery disease and heart failure. However, only a few data exist on miRNAs in patients with arrhythmias. We have previously shown that miR-29b is downregulated in an experimental model of AF and confirmed this downregulation in plasma of AF patients[31]. Interestingly, in our current study miR-29b also showed a trend towards a progressively reduced plasma expression over time. MiR-26a showed a similar expression pattern in plasma in our study (significantly downregulated in elite runners only) and was also shown to be downregulated in human right atrial tissue resulting in an upregulation of the I_K1_ current and a shortening of the action potential duration[36]. In sum, we observed a more moderate and slower expressional response of miR-29b/-26a compared to other miRNAs. Therefore, a larger number of runners or measurement of miRNAs at later time points might show this downregulation more clearly. Another miRNA involved in regulation of the I_K1_ current is miR-1, which has been shown to be upregulated in our study cohort as well as in several animal models[37, 47]. In sum, the expression levels of these miRNAs observed in our cohort are confirmed by studies in patients and in experimental animal models that demonstrated a role of these miRNAs in cardiac remodeling. This further supports circulating miRNAs as potential biomarkers for cardiac remodeling.

### MiRNA abundance in plasma

We have observed a low abundance of miRNAs in plasma (Table 2). This raises potential concerns regarding the validity and reproducibility of our results. Previous studies have also reported low miRNA expression in plasma but the reports generally do not include raw CT values, instead focusing on relative expression levels[1, 41, 48] or fold-changes[2, 4, 40]. These do not allow for direct comparison of results across studies. Of the few available studies that report raw CT values, results are consistent with our present work. Wang et al. evaluated circulating miRNAs as biomarkers for myocardial infarction and reported mean CT values for miR-1 (35.29±0.79, MEAN±SEM) and miR-133a (33.68±0.33, MEAN±SEM)[3]. Nielsen and collegues investigated plasma miRNAs in response to acute exercise and endurance training and demonstrated mean CT values for miR-1 (32.9±1.9, MEAN±SD), miR-29b (32.9±1.8, MEAN±SD), and miR-133a (34.8±1.6, MEAN±SD)[49]. Therefore expression levels of circulating miRNAs are consistently low but within a comparable range indicating valid and reproducible measurements.

To increase confidence in our data, we have reported the range of raw CT data per group. Furthermore, we repeated miRNA measurements at different time points and analyzed the data in a paired manner. This allows consideration of each subject individually over time and facilitates reliable detection of significant differences despite low miRNA abundance.

### Circulating miRNAs in athletes

A small number of studies have been published that report circulating miRNA levels in athletes[40, 41, 48–50]. Baggish et al. performed a study on marathon runners evaluating miRNAs enriched in skeletal muscle (miR-1, miR-133a, miR-499), heart (miR-208a), and vascular endothelium (miR-146a)[40]. All miRNAs analyzed were significantly upregulated after the marathon and returned to baseline 24 hours later. Mooren and colleagues analyzed a similar set of miRNAs in marathon runners and could show a similar pattern of miRNA regulation with a significant increase after the marathon and a decrease 24 hours later for miR-1, -133a, -499, -206, and -208a[41]. Additionally, they showed that miR-21 and miR-155 were not affected by exercise. Our miRathon study confirms the expression profile of miR-1 and miR-133a in an independent third cohort of marathon runners.

Conflicting data were presented by Nielsen et al. who analyzed circulating miRNAs after an acute exercise bout by ergometer cycling and after 12 weeks of endurance training[49]. They demonstrated a general decrease of miRNAs immediately after an acute exercise bout and an upregulation one hour later. MiR-1 (significantly) and miR-133a (non-significantly) showed delayed upregulation 3 hours after the exercise bout only. However, it is doubtful if these results can be compared to results obtained in marathon runners since Banzet et al. showed that exercise modality has a significant impact on miRNA plasma profile[48]. They measured miRNAs over time in volunteers performing two 30-minute walking exercises, either downhill (eccentric exercise) or uphill (concentric exercise)[48]. MiR-1, miR-133a, miR-133b, and miR-208b were not affected by concentric exercise but were significantly upregulated two to six hours after eccentric exercise. Concentric exercise, however, was associated with significant increase of miR-181b and -214, whereas miR-208a was undetectable. The influence of exercise modality on circulating miRNAs was further confirmed by Uhlemann and co-workers[50]. They measured miR-126 and miR-133a in healthy volunteers performing different exercises. A maximal symptom-limited exercise test and four hours bicycling resulted in a significant increase of miR-126 whereas miR-133a was unchanged. Resistance exercise, another eccentric form of exercise, was associated with a miR-133a increase confirming the result ofBanzett. Interestingly, marathon running was another exercise modality tested by Uhlemann et al. and resulted in a significant increase of both miR-126 and miR-133a.

In sum, our study confirms some of the prior observations concerning miRNA biology inendurance athletes. Interestingly, we also identified differences among marathon runners: changes in miRNA plasma expression were more pronounced in elite runners and correlated with LA diameter only in elite runners.Our results suggest that training intensity (elite vs. non elite runners) affects the degree of miRNA expression. This may explain recent findings by Khan et al. who observed that improved fitness is protective of AF only within a certain range, beyond which the risk of AF rises again[51]. What remains unclear is the mechanism by which training intensity affects miRNA plasma expression. A recent study by Padrao et al. demonstrated significant differences in the proteome signaturewith regard to training intensity[52]. Chronic endurance exercise upregulates the tricarboxylic acid cycle and oxidative phosphorylation system while a single bout of exercise affects calcium homeostasis and amino acid metabolism. These changes in energy metabolism provide a potential mechanism for our observations as mitochondrial (dys)function has been shown to affect miRNA expression[53]. It is also possible that exerciseintensity related changes in endothelial function[54] or hemodynamics[55] imposed on the hearts of elite compared to non-elite athletes play a key role in our observations.

### Hemolysis and miRNA plasma levels

It has been shown by Kirchner and colleagues that hemolysis can affect the level of miRNAs circulating in plasma[45]. In their study they evaluated miR-16 and miR-451 and observed a clear correlation between the plasma levels of these two miRNAs and the degree of hemolysis as determined by absorbance measurement at 414 nm. They concluded that hemolysis results in an increase of these two miRNAs. Running a marathon is associated with hemolysis[56]. In fact, measuring absorbance in 13 participants indicated hemolysis in some of the samples (Table 3). However, we think that hemolysis did not affect miRNA levels in our study for several reasons. First, we observed a significant upregulation of miR-1 and miR-133a at V3 and identified correlation of clinical parameters with these two miRNAs. These miRNAs are known to be (skeletal and heart)muscle-specific[57], a fact that is further supported by Doss et al.[58] who performed short RNA transcriptome analysis on human erythrocytes and could not detect miR-1 or miR-133a. They were able to detect miR-26a and miR-29b in erythrocytes, but in our study these miRNAs were downregulated over time. Although we cannot exclude a high clearance rate resulting in paradoxically lower plasma levels it is unlikely that hemolysis is a significant contributor to the plasma levels of miR-26a/miR-29b. Second, we could not detect a correlation between the degree of hemolysis (as indicated by Absorbance at 414nm) and the miRNA levels (Fig 2).

### Release of miRNAs into the circulation

To date, the origin of circulating miRNAs that are measured in the plasma remains unclear. As extreme exercise like marathon running is associated with dehydration it is possible that a miRNA increase after the marathon is a false positive result due to plasma contraction. In our study, however, we used a synthetic cel-miR-39 as spike-in control to normalize for the miRNA content. Furthermore, if plasma contraction influenced our measurement, we would expect to see the plasma levels of miR-26 and miR-29b increase, too. However, miR-26 and miR-29b decreased after the marathon. Therefore, our study results represent a true upregulation of miR-1, miR-30a and miR-133a in plasma of marathon runners.

Another potential explanation for miRNA increase in plasma is the release of miRNAs by destroyed cells. In fact, in our study cohort a significant increase of creatine kinase and troponin was identified after the marathon showing muscle damage[42, 43]. Additionally, we could show that miR-1 and miR-133a plasma levels correlated with CK, CK-MB and/or troponin levels. Therefore, release into the plasma by destroyed cells is a potential explanation for miRNA upregulation. However, several aspects do not support this hypothesis. First, CK and CK-MB plasma levels showed a further increase 24 hours after the marathon (indicating an ongoing cell damage) whereas miR-1 and miR-133a returned to baseline levels. Second, miR-30a showed a similar expression pattern as miR-1 and miR-133a but did not correlate with CK or troponin levels. Third, miR-26a and miR-29b that are also expressed in skeletal muscle and heart[31, 36] are decreased suggesting that cell destruction is not the origin of circulating miRNAs in our study although this could also be due to different clearance rates of these miRNAs. It is possible that excretion is increased or that miRNAs are incorporated into remote cells as indicated by some authors[59, 60]. All these potential mechanisms do not necessarily affect every miRNA to the same degree and could therefore explain different expression patterns over time.

### Role of the left ventricle (LV)

**Fig 9:**
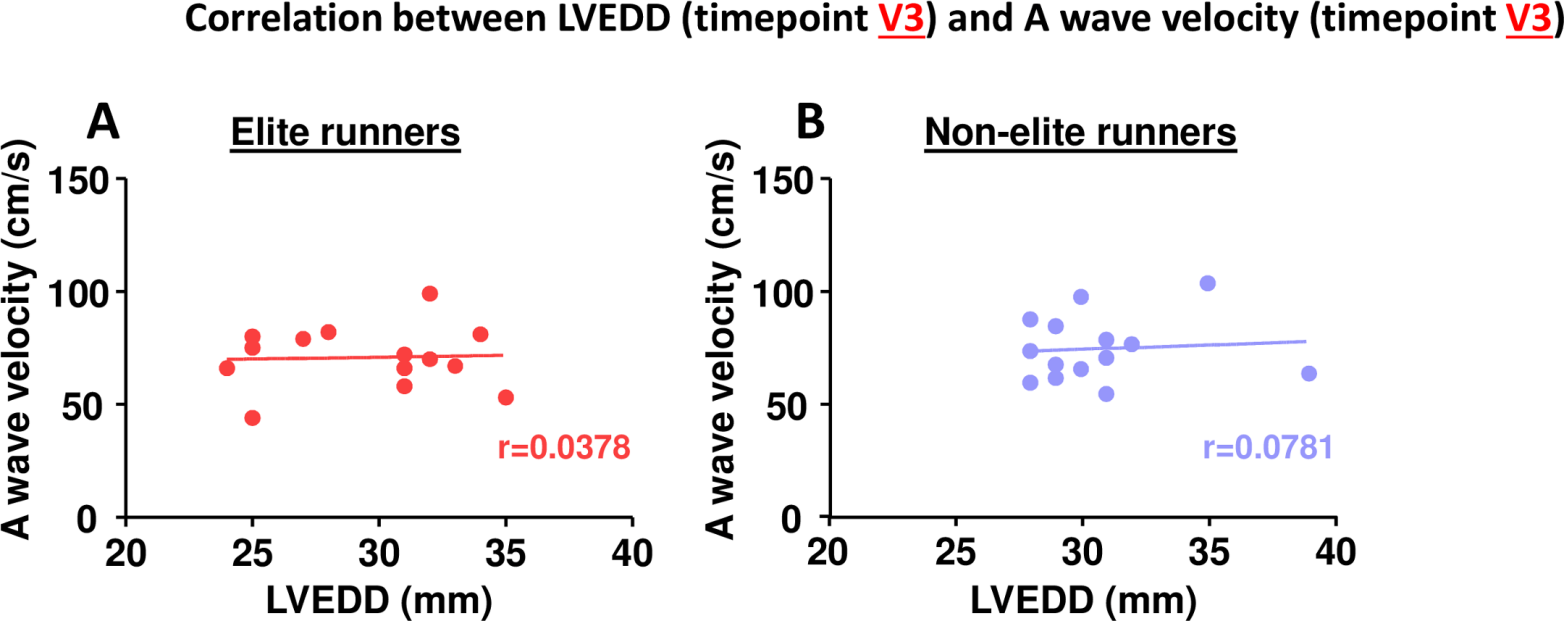
Correlation between LVEDD at timepoint V3 and A wave velocity at timepoint V3. A elite runners, B non-elite runners. Spearman correlation coefficient.

In our study we demonstrate a significant decrease in left ventricular end-diastolic diameter (LVEDD) and a significant increase in A wave velocity after the marathon (V3 vs. V1). One could postulate that the dehydration caused by running a marathon leads to reduced blood volume and preload (manifested by a reduced LVEDD). The increased A wave velocity would therefore simply be a consequence of atrioventricular mechanical coupling, implying that the observed changes in atrial parameters are secondary to global (hydration status) and/or local ventricular changes (LVEDD) rather than independent surrogate markers for atrial remodeling. However, we think this is not true for several reasons. First, this potential relationship between LVEDD and A wave velocity was not shown by other studies evaluating marathon runners. Manier et al. found significant reductions in LVEDD after a marathon but an unchanged A wave velocity[61] whereas Neilan and colleagues observed an unchanged LVEDD and significantly increased A wave velocity[62] suggesting that these two parameters are independent of each other. Second, studies on hemodialysis patients showed that LVEDD is significantly reduced after hemodialysis (i.e. after volume depletion) whereas A wave velocity remains unchanged[63, 64]. Third, these studies also showed that LA volume is significantly reduced after hemodialysis. In contrast, LA diameter was unchanged in our study after the marathon[63, 64]. Finally, LVEDD and A wave velocity did not correlate with each other in our study (Fig 9) suggesting that altered atrial measurements are truly indicative of atrial remodeling and correlating them with miRNA plasma levels is a valid approach.

### Novelty

The miRathon study is the first study evaluating plasma levels of circulating miRNAs in regard to atrial remodeling. The few studies published on circulating miRNAs in athletes to date were either purely descriptive[40, 48–50] or were designed to evaluate miRNAs as potential biomarkers for exercise capacity[41].

Our hypothesis was that circulating miRNAs are biomarkers of atrial remodeling in athletes. Interestingly, we found distinct patterns specific for either elite runners or non-elite runners. First of all, non-elite runners showed less prominent peak levels of miR-1, miR-30a and miR-133a compared to elite runners. The miRNA plasma levels after the marathon correlated with LA diameter only in elite runners, whereas non-elite runners did not show any correlation.This suggests that training intensity (elite vs. non elite runners) affects the degree of miRNA expression and may therefore explain the discrepancy between beneficial moderate physical activity and harmful endurance sports.

Taken together our data suggest that circulating miRNAs can potentially serve as biomarkers of pro-arrhythmogenic signaling leading to structural changes of the atrium in the long term after endurance exercise.

### Potential limitations

In the miRathon study we measured miRNAs in 30 marathon runners, a relatively small number. However, this is the largest cohort of athletes for whom circulating miRNAs have been evaluated so far.

We measured miRNA levels by real-time quantitative PCR (qPCR). Despite all the advantages of this method some limitations especially in regard to miRNA quantification remain. The major disadvantage of this approach is the necessity of a specific probe set for each individual miRNA. Therefore, only a group of miRNAs can realistically be measured (in our study 5 miRNAs). Also, only known miRNAs can be measured. Recently, miRNA quantification by sequencing has emerged as a more comprehensive approach since it allows detection of known and unknown miRNAs as well as isomiRs, and quantification of a large panel of miRNAs at the same time in a high-throughput manner. However, this method is expensive, requires specialised “core facilities” or companies, and data analysis by experienced bioinformaticians[65, 66]. Within the constraints of our study we therefore decided to pursue a candidate-miRNA approach using qPCR.

AF is a chronic disease that develops over decades and has the highest prevalence in people older than 60 years. The average age of our study cohort was 40 years and the observation period was only 11 weeks. Therefore, we did not expect to observe an arrhythmic endpoint like new onset AF. Therefore we focused on surrogate parameters of atrial remodeling. Although these parameters are valuable and widely used they remain surrogates and as such they are not infallible. However, these echocardiographic parameters are the best non-invasive parameters for atrial remodeling currently available. In sum, our data are not evidence of a causal link between circulating miRNAs and development of disease but should rather be seen as an indicator of acute cardiac adaptation in response to exercise. We cannot draw firm conclusions on long-term effects and we do not know if the changes we observed are harmful or whether any of our study participants will develop arrhythmias in the future.

## Conclusion

In our study we observed a characteristic differential expression of circulating miRNAs in athletes and identifiedspecific miRNA expression patterns dependent upon training intensity: we observed a significant correlation between miRNA plasma levels and LA diameter in elite runners only. These are hypothesis-generating and do not prove a direct causal link between circulating miRNA levels and development of AF in athletes. They may however be seen as a potential indicator of atrial remodeling that may or may not result in future disease. Long term follow-up studies are necessary to provide definitive evidence.

## Supporting Information

S1 File **Fig A**. MicroRNA plasma expression over time—Raw Data.Data shown as mean miRNA expression±SEM. *V1 Baseline*, *V2 after 10 weeks training*, *V3 directly after the marathon*, *V4 24h post marathon*. **Fig B**. Correlation between miRNA plasma expression and absorbance at 414 nm–Raw Data. Data shown as absorbance at 414 nm (left column) and miRNA expression (right columns). *V1 Baseline*, *V2 after 10 weeks training*, *V3 directly after the marathon*, *V4 24h post marathon*. **Fig C**. Echocardiographic parameters over time–Raw Data. Data shown as mean±SEM. *LVEDD Left ventricular end diastolic diameter*, *PW posterior wall*, *LA left atrium*, *V1 Baseline*, *V2 after 10 weeks training*, *V3 directly after the marathon*, *V4 24h post marathon*. **Fig D**. Correlation between miRNA plasma expression (V3) and LA diameter (V3).Data shown as miRNA expression (left column) and LA diameter (right column). **Fig E**. Correlation between miRNA plasma expression (V3) and LA diameter (V4). Data shown as miRNA expression (left column) and LA diameter (right column). **Fig F**. Correlation between miRNA plasma expression (V3) and CK plasma levels (V3). Data shown as miRNA expression (left column) and CK plasma levels (right columns). **Fig G**. Correlation between miRNA plasma expression (V3) and CK plasma levels (V3). Data shown as miRNA expression (left column) and CK-MB plasma levels (right column). **Fig H**. Correlation between miRNA plasma expression (V3) and CK plasma levels (V3). Data shown as miRNA expression (left column) and troponin plasma levels (right column). **Fig I**. Correlation between LVEDD (V3) and A wave velocity (V3). Data shown as LVEDD (left column) and peak A wave velocity (right column).*LVEDD Left ventricular end diastolic diameter*.
(XLSX)

## Abbreviations

AF: Atrial Fibrillation
AUC: Area under the curve
miRNA: microRNA
mRNA: messenger RNA
UTR: untranslated region
IAT: individual anaerobic threshold
CI: Confidence Interval
CK: creatine kinase
EF: ejection fraction
LV: left ventricle
LA: left atrium
LVEDD: left ventricular end-diastolic diameter
PW: posterior wall
